# Quality of life and burnout among faculty members: How much does the field of knowledge matter?

**DOI:** 10.1371/journal.pone.0214217

**Published:** 2019-03-22

**Authors:** Priscila Castro Alves, Aurea de Fatima Oliveira, Helena Borges Martins da Silva Paro

**Affiliations:** 1 Department of Public Servants’ Quality of Life and Health, Federal University of Uberlandia, Uberlandia, Minas Gerais, Brazil; 2 Institute of Psychology, Federal University of Uberlandia, Uberlandia, Minas Gerais, Brazil; 3 Department of Humanities in Health, Department of Obstetrics and Gynecology, Federal University of Uberlandia, Uberlandia, Minas Gerais, Brazil; Indiana University, UNITED STATES

## Abstract

**Background:**

Faculty members face demands such as research, outreach programs, and management activities. Such demands may expose faculty to burnout. Burnout affects the physical, psychological and social health of faculty members, but it is still unclear how it affects their quality of life. We aimed to assess the impact of burnout on the quality of life (QoL) of faculty members from different fields of knowledge.

**Methods:**

Cross-sectional study using validated tools for measuring burnout and QoL (*Oldenburg Burnout Inventory*–OLBI and *World Health Organization Quality of Life-Abbreviated version*–WHOQOL-Bref) in a sample of 366 faculty members from a public university. Scores were analyzed using Student’s t-test, analysis of variance (ANOVA), binary logistic regression, and structural equation modeling (SEM).

**Results:**

More than a third of the faculty members (n = 127; 36.6%) suffered from burnout. Men had higher scores of quality of life than women in the physical health (p = 0.001; d<0.5), psychological (p = 0.001; d<0.5) and social relationships (p = 0.048; d<0.5) domains. Women were more exhausted than men (p = 0.001; d<0.5). Faculty members’ perception of quality of life and burnout did not differ according to their field of knowledge (p>0.05). Participants who felt tired before arriving at work were less likely to report good quality of life (OR = 0.46; 95% CI = 0.21–0.99). Faculty members who stated they needed more time to relax after work were less likely to be satisfied with their health (OR = 0.20; 95% CI = 0.10–0.40). Burnout showed a negative association with quality of life (λ = 0.87; p < 0,001; df = 8).

**Conclusions:**

Burnout negatively affects faculty members’ quality of life, regardless of their field of knowledge. Our results suggest the implementation of programs and actions to prevent burnout to faculty members, especially to women, as their quality of life may affect the quality of the education provided.

## Introduction

Working at university as a faculty member may provide satisfactory experiences. However, university teaching can also be stressful and may deteriorate workers’ quality of life [1, 2]. Faculty members face similar pressures to those reported by teachers at other levels of education [3]. However, different demands, such as research, outreach programs, and management activities, are particularly related to faculty work [4]. The effects of this work on faculty members’ health have often been studied, as losses in their quality of life highly affect other people’s lives and the quality of education [5, 6]. Therefore, efforts are necessary to preserve faculty members’ health and quality of life.

Many studies [7–10] argue that a continuous exposure to occupational stress (high work demand and low resources) may cause the burnout syndrome. This syndrome in faculty members is expressed by emotional exhaustion, which is often indicated by attitudes like negative behavior, distancing from students, and negative evaluation of their professional role [11]. Burnout affects the physical, psychological and social health [9] of faculty members, but it is still unclear how it affects their quality of life. Quality of life is closely related to professional life, and it should be assessed in occupational health studies [12].

Studies on quality of life of university faculty members are scarce [2, 4, 13–15]. Among primary and secondary education teachers, studies on quality of life present conflicting evidence regarding gender differences [6, 16, 17]. Some results indicate a lower perception of quality of life among female professionals [6], while other studies show no difference in quality of life according to gender [16, 17]. In studies with university faculty members, there seems to be a relation between mental distress and perception of quality of life among health sciences faculty members [13, 14]. Such studies relating burnout and quality of life have been carried out only among healthcare professionals [13, 14], probably due to the multiple sources of stress in teaching and health care [15]. However, when it comes to faculty members in other fields of knowledge, such as applied human/social sciences and exact/technological sciences, the relation between their quality of life and field of knowledge remains unanswered in the literature.

Our hypothesis is that faculty members’ field of knowledge and burnout may affect their quality of life. We aimed to verify (i) the differences in quality of life and burnout according to faculty members’ gender and field of knowledge and (ii) the association between burnout and faculty members’ quality of life. Identifying groups that have higher risks of presenting worse quality of life may help educational managers to direct strategies towards faculty members’ health and retention.

## Methods

### Participants and study design

This is a cross-sectional study approved by the institutional research ethics committee. This study was held at a public university, with 90 undergraduate programs, 20 PhD programs, 44 academic master’s degree programs, and several other post-graduation programs. Participating faculty members were selected from different academic units and fields of knowledge: applied human/social sciences, life/health sciences, and exact/ technological sciences. As an inclusion criterion, participants should have been working for at least one year in the institution.

When the study was carried out, 1,324 faculty members met the inclusion criterion. In order to determine the differences with a medium effect size (f^2^ = 0.15; p<0.05), with sampling error set at 5% and statistic power at 80%, the sample size comprised 298 participants [18]. Considering a margin of 20% of data loss, we added 75 participants to the sample, adding up to 373 faculty members in the study. Considering the use of structural equations, some authors [19, 20] recommend a sample size between 250 and 500 observations.

Studies have shown that the response rate regarding the study population is between 17% and 35% [4, 21–23]. Thus, in order to reach the size found in the sample calculation, all faculty members that met the inclusion criterion and that were working in the first semester of 2015 (from March to June) were invited to take part in the study (convenience sampling). Faculty members not working during data collection were excluded, i.e. those who were inactive, on vacation or on leave (medical, maternity, educational or private interest leave).

### Procedures

After signing an informed consent, eligible faculty members answered, in their workplace, a self-administered questionnaire with socio-demographic data (age, gender, marital status, working time in the institution, weekly workload, field of knowledge, academic degree), the Oldenburg Burnout Inventory (OLBI), and the World Health Organization Quality of Life-Abbreviated version (WHOQOL-Bref).

OLBI assessed the burnout syndrome in the study population [24]. Its Brazilian version is a psychometrically sound instrument [25] that comprises 9 items, which briefly measure the burnout syndrome. Items are divided into two dimensions: Disengagement (from work) (6 items; α = 0.86) and Exhaustion (3 items; α = 0.76). Exhaustion items approach general feelings of emptiness, overwork, a strong need for rest, and a state of physical exhaustion. Disengagement items involve one’s distancing from the object and content of one’s own work, cynical and negative attitudes and behaviors towards one’s work in general. Answers are rated in a four-point Likert scale, which ranges from 1 (totally disagree) to 4 (totally agree) [24]. Some items have inverted scores, in which 1 means totally agree and 4 totally disagree. Mean scores in the exhaustion dimension higher or equal to 2.25 indicate exhaustion. Scores in the disengagement dimension higher or equal to 2.1 are considered high and denote the presence of disengagement. Individuals with high scores in both dimensions are classified with burnout [26].

The Brazilian validated version of WHOQOL-Bref, a self-administered general questionnaire on quality of life, assessed participants’ quality of life [27]. It comprises 26 items, including two general questions (one approaches self-perception of quality of life, and the other, satisfaction with health), and 24 items divided into four domains: (a) Domain I—Physical health (7 items; α = 0.84): individual’s perception of his/her physical condition; (b) Domain II—Psychological (6 items; α = 0.79): individual’s perception of his/her affective and cognitive condition; (c) Domain III—Social relationships (3 items; α = 0.69): individual’s perception of his/her social relationships and social roles in life; (d) Domain IV—Environment (8 items; α = 0.71): individual’s perception of different aspects related to the environment where he/she lives [27, 28]. The items of the four domains were rated in a five-point Likert scale, with scales of intensity (not at all–extremely), capacity (not at all–completely), frequency (never–always) and assessment (very satisfied–very dissatisfied). Domain scores are rated in a 100-point scale, with higher scores denoting better quality of life [29].

### Statistical analysis

Descriptive statistics (frequency, percentage, mean, and standard deviation) were used to describe the sample, quality of life, and the burnout syndrome. The assumptions of structural equation modeling (SEM) were verified–sample size, normality, missing data, outliers, multicollinearity, singularity, linearity, homoscedasticity [30].

The reliability of each scale in this study was assured through internal consistency (Cronbach’s alpha) [31], with the coefficients of scale domains ranging from 0.76 to 0.88. We compared means according to gender and field of knowledge by applying Student’s *t*-test and analysis of variance (ANOVA). The magnitude of statistically significant differences (effect size) was assessed by determining Cohen’s *d* [32, 33]. The following Cohen’s *d* values were considered small, medium and large effect sizes, respectively: 0.2, 0.5, and 0.8 [32]. Medium effect sizes are often considered clinically important [34].

We also carried out a binary logistic regression analysis to assess which items of the OLBI scale are more probable to affect the general perception of quality of life and satisfaction with health. In this case, OLBI and WHOQOL-Bref answers were dichotomized (0 = disagree, 1 = agree for OLBI; 0 = poor, 1 = good for quality of life, 0 = dissatisfied, 1 = satisfied with health).

We performed structural equation modeling (SEM) to confirm the theoretical framework proposed in this study: the impact of the burnout syndrome in faculty members’ quality of life. In the model, OLBI dimensions (disengagement and exhaustion) behaved as independent variables; and the four domains of WHOQOL-Bref (physical health, psychological, social relationships, and environment) behaved as dependent variables. We used the maximum likelihood method and the traditional chi-square, the chi-square/degrees of freedom ratio (χ^2^/gl), the Root Mean Square Residual (RMR), the Goodness-of-Fit Index (GFI), the Adjusted Goodness-of-Fit Index (AGFI), the Comparative Fit Index (CFI), and the Root-Mean-Square Error of Approximation (RMSEA) as indices of SEM [31, 35].

The significance level was set at 5% for all tests. SPSS Statistics (Statistical Package for Social Sciences) and AMOS (Analysis of Moment Structures) were used for data analysis.

## Results

The final sample comprised 366 participants. Most of them (59.3%) were men, aged between 28 and 69 years (M = 44.8 years; SD = 9.96), and married (74%). They have been working in the institution for 12.3 years (mean) (SD = 11.12). Regarding their field of knowledge, 101 (27.6%) were from exact/technological sciences, 124 (33.9%) from applied human/social sciences, and 141 (38.5%) from health/life sciences. Most participants (85%) had a PhD degree and worked with exclusive dedication to the university (40 hours/week) (86.1%) (Table 1). According to the univariate outliers, 19 cases with score Z > 3.29 (p<0.001) [30] were excluded, totalizing 347 participants. From the total of 347 faculty members, 127 (36.6%) were considered to have burnout.

**Table 1:** Participants’ socio-demographic characteristics, WHOQOL-Bref mean and OLBI classification.

Study variables			
Gender, n(%), N = 366	Male	217	(59.3)
	Female	148	(40.4)
	Not answered	1	(0.3)
Marital status, n(%), N = 366	Single	53	(14.5)
	Married/stable union	271	(74.0)
	Widowed	3	(0.8)
	Divorced	39	(10.7)
Mean age, years (SD)		44.8	(9.96)
Years working in the instituition, mean (SD)		12.34	(11.12)
Field of knowledge, n(%), N = 366	Exact/technological sciences	101	(27.6)
	Human/social sciences	124	(33.9)
	Health/life sciences	141	(38.5)
Week workload, n(%), N = 366	20 hours	5	(1.4)
	40 hours	45	(12.3)
	40 hours (exclusive dedication)	315	(86.1)
	Not answered	1	(0.3)
Degree, n(%), N = 366	Specialization	13	(3.6)
	Mater'sdegree	42	(11.5)
	PhD Degree	233	(63.7)
	Post-doctorate	78	(21.3)
WHOQOL-Bref domain, mean (SD), N = 347	Physical health	71.13	(16.81)
	Psychological	71.63	(15.69)
	Social relationships	67.40	(19.17)
	Environment	65.10	(13.87)
WHOQOL-Bref(1)—"How would you rate your quality of life?" n(%), N = 347	Very poor	0	(0.0)
	Poor	18	(5.2)
	Neither poor nor good	53	(15.3)
	Good	213	(61.4)
	Very good	62	(17.9)
	Not answered	1	(0.3)
WHOQOL-Bref(2)—"How satisfied are you with your health?" n(%), N = 347	Very dissatisfied	1	(0.3)
	Dissatisfied	43	(12.4)
	Neither satisfied nor dissatisfied	64	(18.4)
	Satisfied	184	(53.0)
	Very satisfied	55	(15.9)
OLBI classification, n(%), N = 347	Without burnout	102	(29.4)
	Disengagement	27	(7.8)
	Exhaustion	91	(26.2)
	With burnout	127	(36.6)

### Quality of life and occupational burnout according to gender and field of knowledge

Male participants presented higher scores for quality of life in the physical health, psychological and social relationships domains (p<0.05; 0.22≤*d*≤0.45). Women were more exhausted (p = 0.001; *d* = 0.47) (Table 2). No significant difference was found between faculty members from different fields of knowledge (exact/technological sciences, applied human/social sciences, and health/life sciences) (p>0.05) (Table 3).

**Table 2:** Mean scores of quality of life and occupational burnout according to gender (n = 347).

Domains	Male (n = 207)		Female (n = 139)		*t*	p	*d*^†^
	M	SD	M	SD			
*Physical health*	73.64	15.72	67.62	17.62	3.33	0.001	0.36
*Phychological*	74.50	13.44	67.57	17.66	4.13	0.001	0.45
*Social relationships*	69.16	18.35	65.02	19.99	1.99	0.048	0.22
*Environment*	65.59	13.70	64.73	13.56	0.58	0.565	-
*Disengagement*	2.07	0.58	2.09	0.65	-0.23	0.817	-
*Exhaustion*	2.31	0.69	2.64	0.74	-4.29	0.001	0.47

*t* test; df = 344; *d*^† =^ Cohen’s *d*

**Table 3:** Mean scores of quality of life and occupational burnout according to the field of knowledge (n = 347).

Domains	Exact/technological sciences (n = 96)		Human/social sciences (n = 114)		Health/life sciences(n = 137)		F	p
	M	SD	M	SD	M	SD		
*Physical health*	72.54	16.12	68.73	17.74	72.13	16.40	1.75	0.175
*Phychological*	73.00	14.46	70.94	15.65	71.23	16.57	0.52	0.595
*Social relationships*	67.45	19.42	66.12	19.15	68.43	19.08	0.45	0.637
*Environment*	62.86	12.07	66.10	13.80	65.85	14.98	1.76	0.174
*Disengagement*	2.03	0.62	2.02	0.59	2.17	0.61	2.27	0.105
*Exhaustion*	2.34	0.70	2.50	0.74	2.47	0.74	1.48	0.230

ANOVA; df = 2.344

### Quality of life and occupational burnout: The study model

Concerning the general perception of quality of life, participants who agreed with the items “There are days when I feel tired before I arrive at work” and “After work, I tend to need more time than in the past in order to relax and feel better” were less likely to report good quality of life (OR = 0.46; 95% CI = 0.21–0.99; p = 0.048; OR = 0.28; 95% CI = 0.12–0.63; p = 0.002; respectively). Concerning the general perception of health, faculty members who agreed with the item “After work, I tend to need more time than in the past in order to relax and feel better” were also less likely to report satisfaction with their health (OR = 0.20; 95% CI = 0.10–0.40) (Table 4).

**Table 4:** Binary logistic regression analysis between occupational burnout and the two general questions on quality of life (n = 347).

Variable	QoL N/Total(%)		OR [95%CI]	Health N/Total(%)		OR [95%CI]
	Poor	Good		Dissatisfied	Satisfied	
Over time, one can become disconnected from this type of work.	34/71(47.9)	59/275(21.4)	0.60[0.21–1.70]	53/108(49.1)	41/239(17.1)	1.11[0.44–2.76]
I feel more and more engaged in my work.	33/71(46.5)	195/275(70.9)	1.07[0.43–2.65]	49/108(45.4)	179/239(74.9)	1.44[0.66–3.17]
Lately, I tend to think less at work and do my job almost mechanically.	32/71(45.1)	52/275(18.9)	0.67[0.31–1.47]	44/108(40.7)	41/239(17.1)	1.02[0.49–2.14]
I find my work to be a positive challenge.	49/71(69.0)	248/275(90.2)	2.28[0.96–5.37]	77/108(71.3)	220/239(92.0)	2.11[0.92–4.85]
It happens more and more often that I talk about my work in a negative way.	30/71(42.2)	44/275(16.0)	0.67[0.30–1.50]	42/108(38.9)	33/239(13.8)	1.00[0.46–2.16]
Sometimes I feel sickened by my work tasks.	47/71(66.2)	82/275(29.8)	0.72[0.32–1.62]	67/108(62.0)	63/239(26.4)	0.68[0.33–1.43]
There are days when I feel tired before I arrive at work.	55/70(78.6)	116/275(42.2)	0.46[0.21–0.99]*	77/107(72.0)	95/239(39.7)	0.81[0.42–1.59]
After work, I tend to need more time than in the past in order to relax and feel better.	57/71(80.3)	103/275(37.4)	**0.28[0.12–0.63**]**	86/108(79.6)	75/239(31.4)	**0.20[0.10–0.40**]**
After my work, I usually feel worn out and weary.	52/71(73.2)	107/275(38.9)	1.03[0.46–2.31]	78/108(72.2)	82/239(34.3)	0.83[0.41–1.68]

** p ≤ 0,01

* p ≤ 0,05; OR = odds ratio; CI = confidence interval; QoL = quality of life

**Fig 1:**
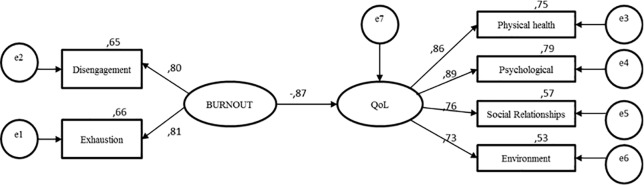
Structural equation modelling: Impact of burnout in faculty members’ quality of life. Parameters: *X*^2^ = 30.962; *X*^2^/df = 3.84; RMR = 0.012; GFI = 0.972; AGFI = 0.926; CFI = 0.982; RMSEA = 0.091; p<0,001; df = 8.

We confirmed the theoretical framework proposed in this study using the maximum likelihood method. Burnout showed a significant negative association with quality of life (λ = 0.87; p < 0.001; df = 8) (Fig 1). The model was considered adequate according to the following parameters: *X*^2^ = 30.962; *X*^2^/df = 3.84; RMR = 0.012; GFI = 0.972; AGFI = 0.926; CFI = 0.982; RMSEA = 0.091.

## Discussion

This study confirms that work issues affect faculty members’ quality of life. This is a pioneer study in approaching the effect of the burnout syndrome on the quality of life of faculty members from different fields of knowledge.

Most participants were satisfied with their quality of life and health. Satisfaction refers to a positive general and emotional state. Faculty members’ affective commitment is closely related to job satisfaction and involvement [36, 37], which, in turn, may be associated with student engagement and outcomes [38]. Faculty satisfaction is also associated with student retention [39]. In this sense, faculty satisfaction may influence the success of the institution in providing significant education to students. The possibility to carry out outreach studies and works with the community, students’ and society’s recognition, gratefulness for contributing to training, autonomy, flexibility, and possibility of learning are some factors possibly related to faculty members’ satisfaction and self-fulfillment [40]. To have good quality of life, faculty members understand it is essential to develop healthy social interactions; to have time to carry out activities and be with their family; to keep healthy habits, such as eating and sleeping well. In addition, faculty members also find important to have some material assets, such as a house, private transportation, adequate work and income, life insurance, among other assets [15].

In our study, female participants had a lower perception of their quality of life in the physical health, psychological, and social relationships domains in comparison to their male peers. They were also more exhausted than male faculty members were. Studies have shown that men and women show different risk behaviors, and men seem to give less importance to their physical and psychological symptoms when compared to women [41]. Based on these considerations, women may answer more critically to instruments that assess quality of life and burnout. Gender differences concerning burnout can also arise from the different strategies used by men and women to face stress [42]. Female faculty members may also perceive more imbalance between work and home as they historically have more child rearing and home-making responsibilities than men do [43]. The dominance of men in the university environment may be another explanation for such results [21, 44–46], which lead to a reflection on the role of cultural stereotypes concerning gender in universities. Sexism and the false idea that science is a male privilege are still noticeable in the academic community [47]. Further, as management and power positions in the university are predominantly occupied by men, individual performance is not the main key for career advancement for women [47]. These facts could contribute to a lower perception of quality of life and burnout among female faculty members.

No significant difference was found among faculty members from different fields of knowledge. Perhaps, the institutional work environment and faculty members’ relation with work have a direct effect on their quality of life, regardless of the field of knowledge or particularities of the work process. The high levels of burnout among faculty members may be another explanation for these results. More than a third of participants suffered from burnout. This result was already expected, since studies carried out in Portugal, Germany, Spain, Mexico, Colombia, United States, and Canada have shown that the prevalence of the burnout syndrome among faculty members is high [46, 48–53], ranging from 14% to 40%. An exaggerated dedication to the teaching activity may explain this fact. Work may affect and modify relationships with family and friends, leisure time, and everyday life [2]. Clearly, the institution or workplace cannot take all the blame for those results, because quality of life concerns other aspects of life beyond work. Faculty members’ perception of quality of life may also be influenced by the safety of the work environment, financial issues, and access to health care. Problems regarding the environmental domain also include basic sanitation, education, health care, work environments and healthy leisure, which are a direct consequence of public policy interventions [54].

The structural equation modeling confirms that burnout may negatively affect faculty members’ quality of life. General feelings of work overload, a strong need for rest, and a state of physical exhaustion are considered important risk factors for the general perception of quality of life and satisfaction with health. Faculty members who feel tired and lack energy are the most affected. Those members have more chances of not enjoying life outside work, not seeing a meaning in their lives, and needing constant medical care. In addition, they are unsatisfied with themselves, with their sleep, their physical appearance, their concentration, their ability to carry out daily activities, and their ability to work. They also have more chances of having negative feelings, such as bad humor, despair, anxiety, and depression. Our results corroborate previous research stating a negative effect of burnout on healthcare faculty members’ quality of life [14]. Studies with other professional categories [55–57] have also confirmed burnout as a risk factor associated with a decrease in quality of life.

Quality of life and burnout among faculty members are not solely determined by the intrinsic character of the job. They are also influenced by how their work is organized, how the educational institution deals with their faculty, and how faculty members see their relationship with their institution [58]. Based on this reflection, our results show a concern and indicate the need for interventions to help faculty members suffering from burnout. Preventive measures directed to faculty members not suffering from the syndrome or already exhausted or disengaged are also necessary. Offering childcare benefits, greater flexibility in work schedule, mentoring, and time management training are possible interventions institutions may put forward to prevent burnout [43]. The incidence of burnout may cause serious losses to universities, because it directly affects productivity and the quality of education due to absences from work. The syndrome can also incapacitate faculty members for work because it is related to different types of personal dysfunctions, such as serious psychological and physical disorders [59].

Our research has some methodological limitations. It is a cross-sectional study with a convenience sample from only one public higher education institution, which restricts generalizations of our results to similar institutions. The use of a generic quality of life questionnaire may have limited conclusions about specific aspects related to work. However, this research used valid and reliable tools to investigate the quality of life and burnout among faculty members, which allows replication and comparisons with further studies. In addition, our study confirms the theoretical model on the effect of burnout on faculty members’ quality of life, with practical and important implications for managers. Knowing how burnout relates to quality of life may support and guide more effective health promotion strategies in the work environment of these professionals.

For future research, other methodological designs can be used (with longitudinal studies and probabilistic sampling), by including universities of different legal natures (public and private), inserting other variables to explain quality of life, e.g. personal (control locus, personality factors) and organizational variables (quality of life at work, perceptions of organizational justice, organizational health, social support, and organizational values–important aspects of the institutional culture). The development of future qualitative studies focused on the causes of faculty members’ distress may also contribute to their work, considering individual and social repercussions in their own health and in the quality of the education provided.
